# Novel Mutations in *TARDBP* (TDP-43) in Patients with Familial Amyotrophic Lateral Sclerosis

**DOI:** 10.1371/journal.pgen.1000193

**Published:** 2008-09-19

**Authors:** Nicola J. Rutherford, Yong-Jie Zhang, Matt Baker, Jennifer M. Gass, NiCole A. Finch, Ya-Fei Xu, Heather Stewart, Brendan J. Kelley, Karen Kuntz, Richard J. P. Crook, Jemeen Sreedharan, Caroline Vance, Eric Sorenson, Carol Lippa, Eileen H. Bigio, Daniel H. Geschwind, David S. Knopman, Hiroshi Mitsumoto, Ronald C. Petersen, Neil R. Cashman, Mike Hutton, Christopher E. Shaw, Kevin B. Boylan, Bradley Boeve, Neill R. Graff-Radford, Zbigniew K. Wszolek, Richard J. Caselli, Dennis W. Dickson, Ian R. Mackenzie, Leonard Petrucelli, Rosa Rademakers

**Affiliations:** 1 Department of Neuroscience, Mayo Clinic, Jacksonville, Florida, United States of America; 2 The ALS Centre, Vancouver General Hospital, Vancouver, British Columbia, Canada; 3 Department of Neurology, Mayo Clinic, Rochester, Minnesota, United States of America; 4 Department of Clinical Neuroscience, Medical Research Council (MRC) Centre for Neurodegeneration Research, King's College London, London, United Kingdom; 5 Institute of Psychiatry, King's College London, London, United Kingdom; 6 Department of Neurology, Drexel University College of Medicine, Philadelphia, Pennsylvania, United States of America; 7 Alzheimer Disease Center, Northwestern University Feinberg School of Medicine, Chicago, Illinois, United States of America; 8 Neurogenetics Program, Department of Neurology, The David Geffen School of Medicine at University of California, Los Angeles, California, United States of America; 9 Eleanor and Lou Gehrig MDA/ALS Research Center, Columbia University, New York, New York, United States of America; 10 Department of Medicine, University of British Columbia, Vancouver, British Columbia, Canada; 11 Department of Neurology, Mayo Clinic, Jacksonville Florida, United States of America; 12 Department of Neurology, Mayo Clinic, Scottsdale, Arizona, United States of America; 13 Department of Pathology, University of British Columbia, Vancouver, British Columbia, Canada; The Jackson Laboratory, United States of America

## Abstract

The TAR DNA-binding protein 43 (TDP-43) has been identified as the major disease protein in amyotrophic lateral sclerosis (ALS) and frontotemporal lobar degeneration with ubiquitin inclusions (FTLD-U), defining a novel class of neurodegenerative conditions: the TDP-43 proteinopathies. The first pathogenic mutations in the gene encoding TDP-43 (*TARDBP*) were recently reported in familial and sporadic ALS patients, supporting a direct role for TDP-43 in neurodegeneration. In this study, we report the identification and functional analyses of two novel and one known mutation in *TARDBP* that we identified as a result of extensive mutation analyses in a cohort of 296 patients with variable neurodegenerative diseases associated with TDP-43 histopathology. Three different heterozygous missense mutations in exon 6 of *TARDBP* (p.M337V, p.N345K, and p.I383V) were identified in the analysis of 92 familial ALS patients (3.3%), while no mutations were detected in 24 patients with sporadic ALS or 180 patients with other TDP-43–positive neurodegenerative diseases. The presence of p.M337V, p.N345K, and p.I383V was excluded in 825 controls and 652 additional sporadic ALS patients. All three mutations affect highly conserved amino acid residues in the C-terminal part of TDP-43 known to be involved in protein-protein interactions. Biochemical analysis of TDP-43 in ALS patient cell lines revealed a substantial increase in caspase cleaved fragments, including the ∼25 kDa fragment, compared to control cell lines. Our findings support *TARDBP* mutations as a cause of ALS. Based on the specific C-terminal location of the mutations and the accumulation of a smaller C-terminal fragment, we speculate that *TARDBP* mutations may cause a toxic gain of function through novel protein interactions or intracellular accumulation of TDP-43 fragments leading to apoptosis.

## Author Summary

The abnormal accumulation of disease proteins in neuronal cells of the brain is a characteristic feature of many neurodegenerative diseases. Rare mutations in the genes that encode the accumulating proteins have been identified in these disorders and are crucial for the development of cell and animal models used to study neurodegeneration. Recently, the TAR DNA-binding protein 43 (TDP-43) was identified as the disease accumulating protein in patients with frontotemporal lobar degeneration with ubiquitin inclusions (FTLD-U) and in amyotrophic lateral sclerosis (ALS). TDP-43 was also found in the brains of 20–30% of patients with Alzheimer's disease (AD). Here, we evaluated whether mutations in TDP-43 cause disease in a cohort of 296 patients presenting with FTLD, ALS or AD. We identified three missense mutations in three out of 92 familial ALS patients (3.3%), and no mutations in AD or FTLD patients. All the identified mutations clustered in exon 6, which codes for a highly conserved region in the C-terminal part of the TDP-43 protein, which is known to be involved in the interaction of TDP-43 with other proteins. We conclude that mutations in TDP-43 are a rare cause of familial ALS, but so far are not found in other neurodegenerative diseases.

## Introduction

Transactive response DNA binding protein with a molecular weight of 43 kDa (TDP-43) is a ubiquitously expressed nuclear protein encoded by the *TARDBP* gene, located on chromosome 1p36. TDP-43 was identified as the major disease accumulated protein in ubiquitinated neuronal cytoplasmic (NCI) and neuronal intranuclear inclusions (NII), that define a growing class of neurological diseases, collectively referred to as *TDP-43 proteinopathies* [1]–[5]. These diseases include amyotrophic lateral sclerosis (ALS), frontotemporal lobar degeneration (FTLD) with ubiquitin immunoreactive, tau negative inclusions (FTLD-U) and FTLD with motor neuron disease (FTLD-MND). In TDP-43 proteinopathies, TDP-43 is relocated from the nucleus to the cytoplasm and sequestered into inclusions that are mainly composed of hyperphosphorylated and C-terminally truncated TDP-43 fragments [4],[6],[7]. TDP-43 immunoreactive histopathology has also been reported in 20–30% of patients with Alzheimer's disease (AD), 70% of patients with hippocampal sclerosis (HpScl), 33% of patients with Pick's disease and in a subset of patients with Lewy-body related diseases [8]–[12]. TDP-43 is a highly conserved protein, containing 2 RNA recognition motifs and a C-terminal glycine-rich domain, known to promote protein-protein interactions [13].

TDP-43 can bind to the common microsatellite region (GU/GT)_n_ in RNA and DNA, with proposed functions in transcriptional regulation [13]. Most recent research has focused on the role of TDP-43 in the regulation of exon 9 alternative splicing in the cystic fibrosis transmembrane conductance regulator gene, however, additional targets have been identified and others likely await identification [14],[15]. TDP-43 has also been implicated in microRNA biogenesis [16].

ALS and FTLD-U are etiologically complex disorders with genetic as well as environmental factors contributing to the disease. A positive family history is reported in 5–10% of ALS patients and in up to 50% of FTLD-U patients, often with an autosomal dominant pattern of inheritance [17]–[19]. Mutations in the Cu/Zn superoxide dismutase gene (*SOD1*) account for ∼10–20% of familial and 1–2% of apparent sporadic ALS patients [20]. However, TDP-43 inclusions were not present in *SOD1* mutation carriers, suggesting a distinct disease mechanism in these patients [21]. The genetic basis of FTLD-U is just starting to be understood [19]. Loss-of-function mutations in the gene encoding the secreted growth factor progranulin (*PGRN*) are a major known cause of familial FTLD-U [22],[23], explaining up to 25% of patients worldwide [24]. Other rare genetic causes of familial FTLD-U include mutations in the valosin containing protein gene (*VCP*) and the gene encoding the charged multivesicular body protein 2B (*CHMP2B*), while some families with a combination of FTLD and ALS show genetic linkage to a locus on chromosome 9p [25]–[29].

Since rare missense mutations and multiplications have been identified in genes encoding the major constituents of the pathological deposits in several neurodegenerative diseases, we hypothesized that mutations in *TARDBP* may contribute to the development of TDP-43 proteinopathies. In fact, the first missense mutations in *TARDBP* were recently discovered in 2 autosomal dominant ALS families and 2 sporadic ALS patients, supporting the central role for TDP-43 in disease pathogenesis [30],[31]. A large population-based study further identified 8 different missense mutations in 3 familial and 6 sporadic ALS patients and showed accumulation of a detergent-insoluble TDP-43 protein product of ∼28 kDa [32]. Here, we report on the extensive mutation screening of *TARDBP* in a diverse cohort of clinical and pathological confirmed patients with neurodegenerative diseases characterized by TDP-43 pathology, which led to the identification of 3 additional ALS families with *TARDBP* mutations. We further show accumulation of proteolytic cleaved fragments with a molecular weight of approximately 35 and 25 kDa in lymphoblastoid cell lines derived from *TARDBP* mutation carriers.

## Results

### *TARDBP* Mutation Analyses

**Figure 1:**
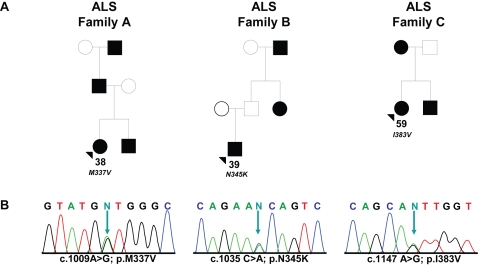
Missense mutations identified in *TARDBP* in familial ALS patients. (A) Pedigrees showing family history of ALS for three probands carrying *TARDBP* mutations. Black symbols represent patients affected with ALS; white symbols represent unaffected individuals. Pedigrees are constructed based on family history data provided by the NINDS Human Genetics Resource Center DNA and Cell Line Repository (http://ccr.coriell.org/ninds). The alive/dead status of individuals is unknown. Arrowheads indicate the probands. The onset age of ALS symptoms and the *TARDBP* mutation identified are included below each proband. (B) DNA sequence traces observed in a sample from the proband of each family. The observed single base substitution and predicted amino acid change are indicated below each chromatogram. cDNA numbering is according to the largest *TARDBP* transcript (NM_007375.3) and starting at the translation initiation codon. Protein numbering is relative to the largest TDP-43 isoform (NP_031401.1).

We performed *in silico* analyses of the *TARDBP* gene structure by alignment of human spliced expressed sequence tags listed in the UCSC genome browser (http://genome.ucsc.edu/). This led to the identification of a novel 5′ non-coding exon (exon 0) in addition to the known non-coding exon 1 and the 5 coding exons that are included in the *TARDBP* reference mRNA sequence (NCBI accession number NM_007375). Sequencing analyses of the 5 coding and 2 non-coding exons of *TARDBP* in our initial cohort of 176 clinical patients and 120 patients with pathologically confirmed TDP-43 pathology revealed 3 heterozygous missense mutations in 3 of the 116 analyzed ALS patients (2.6%), while no mutations were detected in 180 patients affected with FTLD-U, FTLD-MND, AD, HpScl and Lewy-body disease (Table 1, Figure 1). Since all mutation carriers were index patients of autosomal dominant ALS families, the frequency of *TARDBP* mutations increased to 3.3% in the subpopulation of familial ALS patients (3/92 patients). One silent mutation (p.Ala66) and 18 additional sequence variants in intronic and non-coding regions were further identified, none of which was predicted to affect the TDP-43 protein (Table S1). Genomic *TARDBP* copy-number analyses in 208 patients including all 116 ALS patients did not reveal deletions or multiplications.

**Table 1:** Patients included in the *TARDBP* sequencing analyses.

	Patients (N)	Patients with positive family history (N)
**Clinical Diagnoses**		
ALS	95	92
FTLD	60	50
FTLD-ALS	21	11
**Pathological Diagnoses**		
ALS	21	0
FTLD-U	29	25
FTLD-MND	17	8
AD (TDP-43+)	46	21
LBD (TDP-43+)	4	2
HpScl (TDP-43+)	3	1
**Total**	296	210

**Figure 2:**
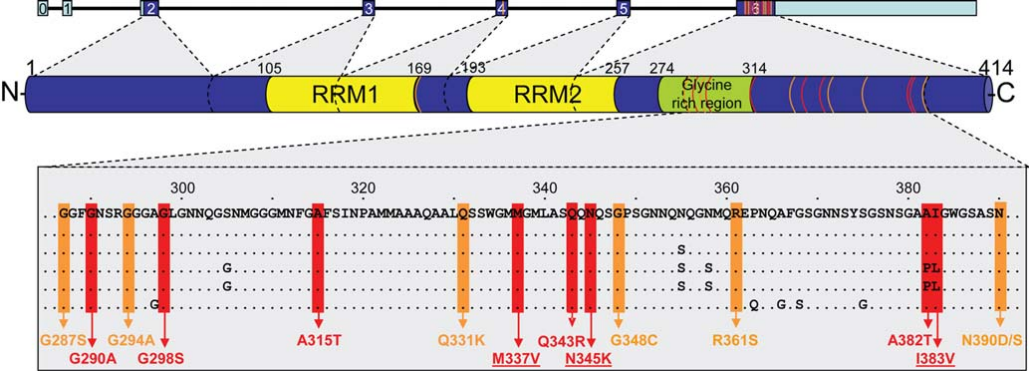
Overview of mutations identified to date in *TARDBP*. Schematic overview of the 7 *TARDBP* exons showing coding regions in dark blue and non-coding regions in light blue (top). The TDP-43 protein structure with location of the conserved domains is shown with protein numbering according to the largest isoforms NP_031401.1 (middle). Protein sequence alignment shows strong conservation in the C-terminal region of TDP-43 (bottom). Colored boxes indicate the position of known and novel TDP-43 mutations identified in sporadic (orange) and familial (red) ALS patients. TDP-43 mutations identified in this study are underlined. Orange and red lines in *TARDBP* gene and TDP-43 protein indicate approximate positions of the mutations. RRM = RNA recognition motif.

All *TARDBP* mutations identified in this study are located in exon 6 (Figure 2). In the index patient of family A (ND10588), we identified the known c.1009 A>G mutation, predicted to substitute valine for methionine at codon 337 (p.M337V), and previously reported to segregate with disease in a large British autosomal dominant ALS kindred. In the index patient of family B (ND08308), a novel mutation c.1035 C>A was identified, predicted to change asparagine to a lysine at codon 345 (p.N345K). Finally, in the index patient of family C (ND08470), a novel mutation c.1147 A>G which predicts an isoleucine for a valine substitution at codon 383 (p.I383V) was identified. Sequence analysis of *TARDBP* exon 6 in 185 healthy control individuals did not identify these or other sequence variants. Using custom made TaqMan genotyping assays, the presence of p.M337V, p.N345K and p.I383V was further excluded in 640 US control individuals. Genotyping 652 sporadic ALS patients for these mutations did not identify additional mutation carriers. Since all 3 mutation carriers were obtained from the National Institute of Neurological Disorders and Stroke (NINDS) Human Genetics Resource Center DNA and Cell Line Repository (Coriell), DNA samples of relatives were unavailable for genetic studies and segregation of the mutations with disease could therefore not be determined.

### Clinical Characteristics of *TARDBP* Mutation Carriers

All 3 *TARDBP* mutation carriers were identified in the clinical patient series and were diagnosed by El Escorial criteria with probable or probable-lab supported ALS. Electromyography (EMG) examination was performed in 2 patients (ND10588 and ND08470) and was supportive of the diagnosis of ALS. A detailed overview of the distribution of upper and lower motor neuron signs in the *TARDBP* mutation carriers is included in Table S2. Patients ND10588 and ND08308 showed early onset ages of 38 and 39 years, respectively, while patient ND08470 showed symptom onset at 59 years (Figure 1). The initial presenting symptom in patients ND10588 and ND08470 was upper-limb ALS, while ND08308 suffered from lower-limb onset ALS. No signs of dementia or other atypical features of ALS were reported in any of the mutation carriers or their affected relatives. No autopsy of *TARDBP* mutation carriers was available.

### Allele Sharing Analyses of *TARDBP* p.M337V

To investigate whether our US p.M337V mutation carrier and the previously reported p.M337V family from the UK are descendants of a common founder, we did an allele sharing study with 12 short tandem repeat (STR) markers spanning a region of 6.7 Mb flanking *TARDBP*, including 5 markers within 1.0 Mb of *TARDBP* (Table 2). We determined the disease haplotype in the UK family and compared this to the genotypes observed in ND10588 to detect allele sharing. Shared alleles were observed for 6 out of 12 STR markers in the region, however, only one marker (Chr1(AC)_11.06) directly flanking *TARDBP* was shared and the 264 bp allele identified at this marker was common in the population (62.4%). In addition, potentially shared alleles at all other markers in the region were also common (>28%). These results make it unlikely that p.M337V originated from a common founder.

**Table 2:** Allele sharing in p.M337V families.

Marker	Genomic position^a^	Linked allele (bp) UK family	Frequency of linked allele (%)	Patient ND10588^b^
D1S2663	7.18	**201**	8.9	189–199
D1S2694	7.26	**238**	50.0	**238**–238
D1S450	9.51	**255**	10.7	249–251
D1S1635	10.91	**160**	14.8	147–157
*c.1009A>G (p.M337V)*	11.01	**G**	-	A–**G**
Chr1_11.06	11.06	**264**	62.4	**264**–270
Chr1_11.28	11.28	**128**	14.0	132–132
D1S2667	11.41	**264**	28.6	260–**264**
D1S2740	11.84	**90**	57.4	**90**–100
D1S489	11.97	**143**	37.5	**143**–143
D1S434	12.25	**240**	1.8	246–248
D1S1597	13.66	**171**	49.5	**171**–171
D1S228	13.86	**121**	26.8	119–123

a Genomic position relative to the UCSC genome browser on the Human Mar. 2006 Assembly (http://genome.ucsc.edu/).

b Alleles that are shared between the UK family and patient ND10588 are in bold.

### Biochemical Analysis of *TARDBP* Mutations in Familial ALS Patients

**Figure 3:**
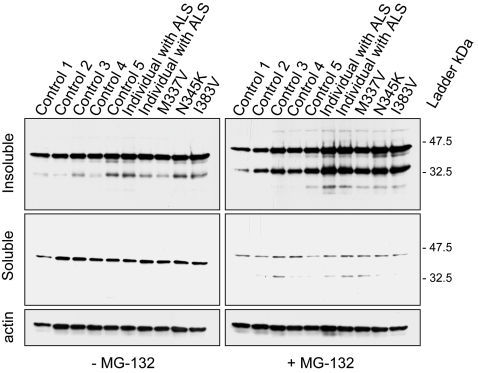
Biochemical analysis of TDP-43 in lymphoblastoid cell lines of *TARDBP* mutation carriers. Western blot analyses of protein lysates derived from lymphoblastoid cell lines from 3 familial ALS patients carrying different *TARDBP* mutations (p.M337V, p.N345K and p.I383V), 2 ALS patients (1 and 2) without *TARDBP* mutations and 5 healthy control individuals (Control 1–5). In lymphoblastoid cell lines derived from *TARDBP* mutation carriers and sporadic ALS patients an accumulation of 2 smaller C-terminal fragments of TDP-43 protein of approximately 35 and 25 kDa was observed in detergent-insoluble fractions treated with the proteasome inhibitor, MG-132. In lymphoblastoid cell lines derived from control individuals the levels of the 35 kDa fragment were substantially lower, and the 25 kDa fragment was mostly undetectable. Membranes from the soluble fraction were reprobed for beta-actin to monitor protein loading.

Kabashi and colleagues previously reported a substantial increase in a ∼28 kDa fragment in lymphoblastoid cells with *TARDBP* mutations in the presence of the proteasomal inhibitor, MG-132, but not in lymphoblastoid cells derived from control individuals or ALS patients suggesting an increase aggregation property of these TDP-43 mutants [32]. Based on this result, we performed a similar study and analyzed the 3 patients with *TARDBP* mutations identified in our study, 2 sporadic ALS cases and 5 control individuals in the presence or absence of MG-132. Consistent with the previous report, a marked increase in the accumulation of detergent insoluble TDP-43 protein fragments were observed in the lymphoblastoid cell lines treated with MG-132 derived from patients with *TARDBP* mutations but not those derived from control individuals. In our study, we sized the higher and lower TDP-43 C-terminal fragments at approximately 35 and 25 kDa respectively (Figure 3). A similar increase was also found in individuals with sporadic ALS (Figure 3).

**Figure 4:**
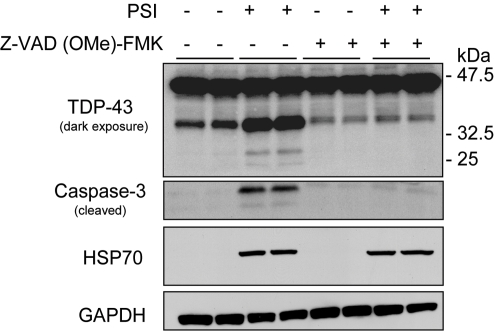
Proteasome inhibition increases the proteolytic cleavage of TDP-43. Western blot analyses of H4 neuroglioma cells treated with the proteasome inhibitor, PSI (10 µM, 24 hours) and a pan-caspase inhibitor, Z-VAD-FMK (100 µM, 24 hours) separately or in combination. Treatment with PSI revealed an increase in proteolytic cleavage of TDP-43 fragments (35 and 25 kDa) and an increase in caspase-3 activity. Treatment with a pan-caspase inhibitor suppressed PSI-induced TDP-43 cleavage and caspase-3 activity. HSP70 levels were increased after PSI treatment and the levels persisted in the presence of a pan-caspase inhibitor. Similar results were obtained in 3 independent experiments.

We previously demonstrated that the proteolytic cleavage of TDP-43 by caspases can generate insoluble C-terminal fragments (35 and 25 kDa) similar to those found in diseased brains. Therefore, we investigated whether proteasome-induced toxicity was associated with proteolytic processing of endogenous TDP-43 in cell culture models. H4 neuroglioma cells were treated with either vehicle (DMSO) or proteasome inhibitor I (PSI) (10 µM) for 24 hours. In the presence of PSI, TDP-43 was cleaved into ∼35 and ∼25 kDa fragments (Figure 4), similar to the 35 and 25 kDa fragments found in the lymphoblastoid cell lines derived from the *TARDBP* mutation carriers (Figure 3). Similar results were obtained using MG-132 (data not shown). The inhibitory activity and toxicity of PSI also led to a marked increase in cleaved (active) capase-3 levels, which promotes apoptotic cell death and accumulates upon such inhibition. Furthermore, when we co-treated the cells with PSI and the caspase inhibitor, Z-VAD (OMe)-FMK, the generation of proteolytic TDP-43 fragments was inhibited (Figure 4). HSP70 immunoblot analysis was used to verify the inhibition of the proteasomal machinery. As expected, HSP70 levels were increased after PSI treatment and the levels persisted in the presence of caspase inhibitor Z-VAD (OMe)-FMK (Figure 4). Taken together, these data strongly suggest that proteasome inhibition is sufficient to promote proteolytic cleavage and accumulation of TDP-43 through a mechanism that implicates programmed cell death.

## Discussion

The identification of rare mutations in genes encoding the major protein component of the pathologic brain depositions observed in familial neurodegenerative diseases has played a critical role in our current understanding of the molecular pathways underlying AD (*APP*), FTLD (*MAPT*) and Parkinson's disease (*SNCA*) [33],[34]. In this study, we performed mutation analyses of *TARDBP*, encoding TDP-43, in a large cohort of patients with neurodegenerative diseases characterized by TDP-43 pathology to determine if rare mutations or multiplications in *TARDBP* are involved in the genetic etiology of TDP-43 proteinopathies. Patients with a clinical diagnosis of ALS, FTLD or FTLD-ALS, and patients with pathologically confirmed TDP-43-proteinopathy were included in the analyses. In support of our hypothesis, 14 different pathogenic *TARDBP* missense mutations were reported by other researchers during the course of this study in familial and sporadic ALS patients [30]–[32],[35].

We identified 2 novel *TARDBP* missense mutations (p.N345K and p.I383V) and the known p.M337V mutation in 3 out of 92 familial ALS patients (3.3%), while no mutations were identified in 24 sporadic ALS patients or 180 patients with other neurodegenerative diseases. p.M337V, p.N345K and p.I383V were excluded in 825 US control individuals and in 652 additional sporadic ALS patients. The *TARDBP* mutation frequency in our familial ALS cohort is comparable to the frequency reported by Kabashi and colleagues [32] (3/80 patients = 3.8%) but considerably higher than the frequency reported by Sreedharan and colleagues (1/154 patients = 0.6%) [31]. This may reflect the difference in study design, as a significant number of our patients were index patients of autosomal dominant ALS families, including all 3 patients carrying *TARDBP* mutations. Unfortunately, since all mutation carriers were index patients obtained from the NINDS Human Genetics Resource Center DNA and Cell Line Repository, DNA of affected relatives was not available to determine segregation of the mutations with disease. The absence of *TARDBP* mutations in patients with neurodegenerative diseases other than ALS in our study, confirms the lack of mutations and genetic association of *TARDBP* in FTLD populations [30], [36]–[38]. However, without extensive *TARDBP* sequence analyses in additional cohorts of FTLD and AD patients, *TARDBP* mutations cannot be excluded as a rare cause of these disorders.

All *TARDBP* mutation carriers identified in this study presented with probable ALS according to El Escorial criteria in the absence of atypical clinical signs, in agreement with the previous reports on *TARDBP* mutation carriers.

The p.M337V mutation has previously been reported to segregate with disease in a British autosomal dominant ALS family [31]. We identified p.M337V in an index patient from a US family with a strong family history of ALS. Our mutation carrier showed upper limb-onset ALS at 38 years of age, 6 years younger than the earliest onset age reported in the British p.M337V family. Signs of dementia were not reported in any of the family members, consistent with the previous report. An allele sharing study using 12 STR markers flanking *TARDBP* did not support a common ancestor for the UK family and our US patient, although our set of analyzed markers would not have detected a very distant common ancestor [39],[40]. In addition, we cannot exclude the rare possibility that marker Chr1_11.28 mutated in patient ND10588 or that the genomic position of this marker is incorrect, which would leave open the possibility of a shared region of maximum 1.3 Mb (D1S1635-D1S434). In anyway, the identification of p.M337V in two genealogically unrelated ALS families adds further strength to the pathogenicity of *TARDBP* mutations and justifies mutation screening for *TARDBP* in patients with familial ALS.

Similar to 13 of the 14 previously reported *TARDBP* mutations, both novel missense mutations identified in this study were located in exon 6 encoding the highly conserved C-terminus of TDP-43, known to be involved in protein-protein interactions (Figure 2). p.N345K was identified in a 43 year old male with a 4 year history of ALS and an autosomal dominant family history. The p.I383V mutation was also identified in a familial ALS patient; however the onset age was 59 years, 2 decades later than the other 2 mutations identified in this study. This may reflect the more conservative amino acid substitution (Iso→Val) or its more C-terminal location in the TDP-43 protein compared to the other mutations, which may induce a different disease mechanism. Alternatively, additional genetic and/or environmental factors may determine the disease expression of *TARDBP* mutations, as suggested by the wide onset age range (48–83 years) observed in the recently published family with the p.A315T mutation in *TARDBP* [30]. Finally, although there is strong evidence supporting that p.N345K and p.I383V are pathogenic, there remains the possibility that these mutations in fact represent rare benign polymorphisms. Definitive confirmation of their pathogenic nature will depend on finding additional ALS patients carrying these mutations.

To determine the pathological significance of *TARDBP* missense mutations on the post-translational processing of TDP-43, we examined human lymphoblastoid cell lines derived from all 3 familial *TARDBP* mutation carriers identified in this study, 2 ALS patients without *TARDBP* mutations and 5 control individuals (Figure 3). Patient cell lines revealed a substantial increase in a proteolytic cleaved fragment with a molecular weight of approximately 35 and 25 kDa consistent with caspase cleavage [7]. These data suggest that *TARDBP* mutations may cause a toxic gain of function through novel protein interactions or intracellular accumulation, particularly of caspase fragments. Kabashi and colleagues previously reported a similar substantial increase in a fragment of approximately 28 kDa in lymphoblastoid cell lines of *TARDBP* mutation carriers. This fragment accumulated in the presence of a proteasome inhibitor (MG-132), which led the authors to speculate that this TDP-43 product is likely degraded by the ubiquitin-proteasome system (UPS) [32]. While we can't exclude the enhanced aggregation of their mutants in the presence of the inhibitor, our data suggests that proteasome-induced toxicity enhances proteolytic cleavage of TDP-43 into 35 and 25 kDa fragments, resulting in cleavage fragments similar to those observed in ALS patients (Figure 4). Although we can't exclude the possibility that these fragments may be degraded by the UPS, it is likely that the accumulation of these fragments is primarily mediated by caspase cleavage.

In conclusion, our findings support that *TARDBP* mutations are a rare cause of ALS, but so far are not found in other neurodegenerative diseases. Since all reported *TARDBP* mutations cluster in exon 6 encoding a highly conserved region of the TDP-43 protein, selective mutation analyses of *TARDBP* exon 6 in familial and sporadic ALS may be warranted.

## Materials and Methods

### Study Populations

Our initial study population comprised a total of 296 patients with TDP-43 related neurodegenerative diseases, including 176 clinically diagnosed patients with ALS, FTLD and FTLD-ALS and 120 patients with pathologically confirmed TDP-43 proteinopathy. The average age at onset in the clinical cohort was 57.8±10.7 (range 31–81 years) and the average age at death in the pathological cohort was 74.8±13.8 (range 38–100 years). Among patients with known ethnicities (N = 214), 95% were Caucasian (N = 203), 3% were Hispanic (N = 7) and 2% were others (African/American (N = 2), East-Indian (N = 1) and Caribbean (N = 1)). A summary of the primary diagnoses and family history of the patients is provided in Table 1. The majority of the pathological confirmed patients (N = 87) were derived from the Mayo Clinic Jacksonville Brain Bank and primarily ascertained through The State of Florida Alzheimer's Disease Initiative funded through the Department of Elder Affairs, The Einstein Aging Study, The Udall Center for Excellence in Parkinson's Disease Research, CurePSP/The Society for Progressive Supranuclear Palsy, the Mayo Alzheimer's Disease Patient Registry (ADPR) and the Florida Alzheimer's Disease Research Center (ADRC). Additional clinical and pathological confirmed patients were ascertained through the Mayo Clinic Jacksonville and Rochester ADRC (N = 60), Mayo Clinic Scottsdale Alzheimer's Disease Center (ADC) (N = 4), the Neurological Institute of New York, Columbia University (N = 2), the University of California, Los Angeles (UCLA) ADC (N = 23), the University of British Columbia (N = 58), the Harvard Brain Bank (N = 5), the Sun Health Research Institute (N = 4), the Drexel University College of Medicine (N = 1), the Northwestern Feinberg School of Medicine (N = 13) and the Coriell Institute for Medical Research (N = 39). A list of the specific samples from the Coriell Institute included in the *TARDBP* mutation screening is provided as Table S3.

To determine the frequency of the *TARDBP* mutations identified in our initial cohort, an additional cohort of 652 sporadic ALS patients was obtained from the University of British Columbia (N = 140), the Neurological Institute of New York, Columbia University (N = 48) and the Coriell Institute for Medical Research (N = 464). All control individuals (N = 825) included in the study were Caucasian and ascertained through the Mayo Clinics in Jacksonville, Florida and Scottsdale, Arizona.

### Sequencing Analysis

The 5 coding and 2 non-coding exons of *TARDBP* were amplified by polymerase chain reaction (PCR) in standard 25 µl reactions using Qiagen PCR products (Table S4). PCR products were purified using the Agencourt Ampure method and sequenced using Big dye terminator V.3.1 products. Sequencing products were purified using the Agencourt CleanSEQ method and analyzed on an ABI 3730 DNA analyzer (Applied Biosystems, Foster City, CA, USA).

### Genotyping

The presence of *TARDBP* mutations c.1009A>G (p.M337V), c.1035 C>A (p.N345K) and c.1147 A>G (p.I383V) in sporadic ALS patients and control individuals was determined with custom-designed TaqMan SNP genotyping assays (Applied Biosystems) (Table S5) and analyzed on an ABI7900 genetic analyzer using SDS2.2.2 software.

### *TARDBP* Copy-Number Analyses

TaqMan gene expression assays to exons 2, 4 and 6 of *TARDBP* and to exon 5 of *PSEN2* (for use as endogenous control) were designed using File Builder 3.1 software (Applied Biosystems) (Table S6) to test for the presence of genomic *TARDBP* copy-number mutations in 208 patients selected from our population. This approach was used to detect copy-number mutations affecting exons 2, 4 or 6, as well as complete *TARDBP* and large N- and C-terminal *TARDBP* deletions and multiplications. Real-time PCR with 25 ng genomic DNA as template was performed on an ABI7900 using the TaqMan method according to standard procedures. All samples were run in triplicate. The FAM-fluorescent signal was analyzed using SDS2.2.2 software, and genomic copy number determined by relative quantification (ΔΔct method).

### p.M337V Allele Sharing Studies

To examine whether the US and UK families carrying the p.M337V mutation shared a common founder, we typed 12 STR markers spanning a region of 6.7 Mb flanking *TARDBP* in 3 patients and 8 unaffected relatives of the previously published UK family, in the US patient ND10588 and in 2 CEPH samples. STR markers were amplified with one fluorescently labeled primer and PCR fragments were analyzed on an automated ABI3100 DNA analyzer. Alleles were scored using the Genemapper software (Applied Biosystems). CEPH allele frequencies were used to estimate the allele frequency of the shared alleles in control individuals (CEPH genotype database; http://www.cephb.fr/cephdb/). The 2 novel markers were PCR amplified using Chr1_11.06-F: FAM-CAGCATCATGTGGTTTGGCAGT, Chr1_11.06-R: CAGCTCGCAGGGAAGATGAAA, Chr1_11.28-F: FAM-TGGCCATCTTAACAGGAACAGC and Chr1_11.28-R:TTCAAGGGCTTTCGAGGTGAA and allele frequencies were estimated in a population of 93 unrelated US control individuals.

### Cell Culture and Treatment

H4 neuroglioma cells were grown in Opti-Mem plus 10% FBS and 1% pen-strep. Cells were plated in 6-well plates and at 90% confluency treated with 10 µM proteasome inhibitor I (PSI) (EMD Chemicals, Inc. San Diego, CA) or 100 µM pan-caspase inhibitor (Z-VAD-FMK) (EMD Chemicals, Inc. San Diego, CA) separately or in combination. Twenty-four hours after treatment, cells were harvested for subsequent Western blot analysis in the Co-IP buffer (50 mM Tris-HCl, pH 7.4, 1 M NaCl, 1% Triton-X-100, 5 mM EDTA) plus 1% SDS, PMSF, protease and phosphatase inhibitors. A similar experiment was performed using 10 µM MG-132 (Calbiochem, San Diego, CA) instead of PSI.

### Fractionation Experiment

Lymphoblastoid cells from 5 healthy control individuals, 3 familial ALS patients with *TARDBP* mutations and 2 ALS patients without *TARDBP* mutations were grown in RPMI1640 plus 10% FBS and 1% pen-strep. Cells were plated in T25 flasks and treated the following day with MG-132 (20 µM, 6 hours). Cell pellets from each cell line were lysed with the 0.2% Triton X-100-PBS with PMSF, protease and phosphatase inhibitors on ice for 10 minutes. After sonication, samples were centrifuged at 10,000 g for 15 minutes at 4°C. The supernatant was saved as the soluble fraction and the pellet was resuspended, sonicated in 2% SDS-PBS-Urea and saved as the insoluble fraction. The soluble and insoluble fractions were subjected to Western blot analysis.

### Western Blot Analysis

Protein concentrations of cells lysates were measured by a standard BCA assay (Pierce, Rockford, IL). Next, samples were heated in Laemmli's buffer and equal amounts of protein were loaded into 10-well 10% or 4–20% Tris-glycine gels (Novex, San Diego, CA). After transfer, blots were blocked with 5% nonfat dry milk in TBST (TPS plus 0.1% Triton X-100) for 1 hour, and then incubated with rabbit polyclonal TDP-43 antibody (1∶500; ProteinTech Group, Inc, Chicago, IL), rabbit polyclonal caspase-3 antibody (1∶1000; Cell Signaling, Beverly, MA), HSP70 (1∶2000; Stressgen, Ann Arbor, MI) or mouse monoclonal β-actin antibody (1∶5000, Sigma, Saint Louis, MS) overnight at 4°C. Membranes were washed three times each for 10 minutes with TBST and then incubated with anti-mouse or anti-rabbit IgG conjugated to horseradish peroxidase (1∶2000; Jackson ImmunoResearch, West Grove, PA) for 1 hour. Membranes were then washed three times each for 10 minutes, and protein expression was visualized by ECL treatment and exposure to film.

## Supporting Information

Table S1 Sequence variants identified in *TARDBP*.
(0.07 MB DOC)

Table S2 Distribution of Upper and Lower Motor Neuron signs in *TARDBP* mutation carriers.
(0.03 MB DOC)

Table S3 Specific samples from the Coriell Institute included in the *TARDBP* mutation analyses.
(0.07 MB DOC)

Table S4 *TARDBP* PCR and sequencing primers.
(0.03 MB DOC)

Table S5 Primers and probes for *TARDBP* copy-number analyses.
(0.03 MB DOC)

Table S6 Detailed Information on *TARDBP* Taqman genotyping assays.
(0.03 MB DOC)
