# Supplementary material for: Novel Mutations in TARDBP (TDP-43) in Patients with Familial Amyotrophic Lateral Sclerosis
Source: PLoS Genet. 2008 Sep 19;4(9):e1000193. doi: 10.1371/journal.pgen.1000193 (PMC2527686; doi:10.1371/journal.pgen.1000193)
Supplement: Table S1 — Sequence variants identified in TARDBP. (0.07 MB DOC) [file pgen.1000193.s001.doc]

***Table S1. Sequence variants identified in TARDBP.***

| **Region** | **Alias** | **Genomic mutation^1^** | **Predicted cDNA^2^** | **Predicted protein^3^** | **dbSNP ID** | **Previously identified** |
| --- | --- | --- | --- | --- | --- | --- |
| 5' region | 5'-147G>A | g.8823G>A |  |  |  | - |
| 5' region | 5'-145delC | g.8825delC |  |  |  | [36] |
| 5' region | 5'-100C>T | g.8870C>T |  |  | rs968545 | [36] |
| 5' region | 5'-21C>T | g.8949C>T |  |  |  | - |
| Exon 1 | Ex1+13G>A | g.8982G>A | c.-122G>A |  | rs11121679 | - |
| Exon 1 | EX1+42G>A | g.9011G>A | c.-93G>A |  |  | - |
| Intron 1 | IVS1+74C>A | g.9165C>A | c.-13+74C>A |  | rs11121680 | - |
| Intron 1 | IVS1+85T>C | g.9167T>C | c.-13+85T>C |  | rs4133584 | [36,37] |
| Intron 1 | IVS1+95C>T | g.9186C>T | c.-13+95C>T |  | rs11121681 | - |
| Intron 1 | IVS1+215C>T | g.9306C>T |  |  |  | - |
| Intron 1 | IVS1+218C>T | g.9309C>T | c.-13+218C>T |  |  | - |
| Exon 2 | Ala66 | g.10273T>C | c.198T>C | p.A66 |  | [30,36] |
| Intron 2 | IVS2-15G>T | g.13177G>T | c.239-15G>T |  |  | - |
| Intron 3 | IVS3+106C>T | g.13461C>T | c.402+106C>T |  |  | - |
| Intron 4 | IVS4+9G>A | g.15230G>A | c.543+9G>A |  |  | - |
| Intron 4 | IVS4+112C>A | g.15333C>A | c.543+112C>A |  |  | [36] |
| Intron 5 | IVS5+70delG | g.17016delG | c.714+70delG |  |  | [36] |
| 3'UTR | 3'UTR+87T>A | g.20125T>A | c.*87T>A |  |  | - |
| 3'UTR | 3'UTR+208C>T | g.20246C>T | c.*208G>A |  |  | [36] |

^1^gDNA numbering relative to GenBank Accession Number AL109811.40 and starting at nucleotide 1

^2^cDNA numbering according to the largest *TARDBP* transcript with GenBank Accession Number NM_007375.3 and starting at the translation initiation codon

^3^Protein numbering according to the largest TDP-43 isoform with GenPept Accession Number NP_031401.1
