# Supplementary material for: Novel Mutations in TARDBP (TDP-43) in Patients with Familial Amyotrophic Lateral Sclerosis
Source: PLoS Genet. 2008 Sep 19;4(9):e1000193. doi: 10.1371/journal.pgen.1000193 (PMC2527686; doi:10.1371/journal.pgen.1000193)
Supplement: Table S2 — Distribution of Upper and Lower Motor Neuron signs in TARDBP mutation carriers. (0.03 MB DOC) [file pgen.1000193.s002.doc]

**Table S2. Distribution of Upper and Lower Motor Neuron signs in *TARDBP* mutation carriers.**

|  |  |  |  |  | **Upper Motor Neuron signs**  **supporting diagnosis** | | | | **Lower Motor Neuron signs**  **supporting diagnosis** | | | |
| --- | --- | --- | --- | --- | --- | --- | --- | --- | --- | --- | --- | --- |
| **Patient ID (mutation)** | **Ethnicity** | **Gender** | **Onset age** | **El Escorial diagnosis** | **Bulbar** | **Cervical** | **Thoracic/ chest** | **Lumbosacral/ lower limbs** | **Bulbar** | **Cervical** | **Thoracic/ chest** | **Lumbosacral/ lower limbs** |
| ND10855 (p.M337V) | Caucasian | F | 38 | Probable | definite | definite | not tested | definite | absent | definite | not tested | definite |
| ND08308  (p.N345K) | Caucasian | M | 39 | Probable | definite | definite | not tested | definite | definite | definite | absent | indeterminate |
| ND08470  (p.I383V) | Caucasian | F | 59 | Probable/ lab supported | absent | absent | absent | absent | absent | definite | definite | indeterminate |
