# Supplementary material for: Novel Mutations in TARDBP (TDP-43) in Patients with Familial Amyotrophic Lateral Sclerosis
Source: PLoS Genet. 2008 Sep 19;4(9):e1000193. doi: 10.1371/journal.pgen.1000193 (PMC2527686; doi:10.1371/journal.pgen.1000193)
Supplement: Table S3 — Specific samples from the Coriell Institute included in the TARDBP mutation analyses. (0.07 MB DOC) [file pgen.1000193.s003.doc]

**Table S3. Specific samples from the Coriell Institute included in the *TARDBP* mutation analyses.**

| **NINDS ID** | **Diagnosis** | **Onset Age** | **Gender** | **Ethnicity** |
| --- | --- | --- | --- | --- |
| ND05367 | AMYOTROPHIC LATERAL SCLEROSIS | 46 | F | Caucasian |
| ND06172 | AMYOTROPHIC LATERAL SCLEROSIS | 43 | M | African American |
| ND06751 | AMYOTROPHIC LATERAL SCLEROSIS | 50 | M | Caucasian |
| ND06880 | AMYOTROPHIC LATERAL SCLEROSIS | 48 | F | Caucasian |
| ND07333 | AMYOTROPHIC LATERAL SCLEROSIS | 60 | M | Caucasian |
| ND07489 | AMYOTROPHIC LATERAL SCLEROSIS | 66 | M | Caucasian |
| ND08308 | AMYOTROPHIC LATERAL SCLEROSIS | 39 | M | Caucasian |
| ND08470 | AMYOTROPHIC LATERAL SCLEROSIS | 59 | F | Caucasian |
| ND08957 | AMYOTROPHIC LATERAL SCLEROSIS | 46 | M | Caucasian |
| ND09014 | AMYOTROPHIC LATERAL SCLEROSIS | 50 | M | Caucasian |
| ND09190 | AMYOTROPHIC LATERAL SCLEROSIS | 72 | F | Caucasian |
| ND09288 | AMYOTROPHIC LATERAL SCLEROSIS | 51 | F | Caucasian |
| ND09373 | AMYOTROPHIC LATERAL SCLEROSIS | 56 | M | Caucasian |
| ND09492 | AMYOTROPHIC LATERAL SCLEROSIS | 51 | M | Caucasian |
| ND09607 | AMYOTROPHIC LATERAL SCLEROSIS | 52 | M | Caucasian |
| ND09677 | AMYOTROPHIC LATERAL SCLEROSIS | 69 | F | Caucasian |
| ND09786 | AMYOTROPHIC LATERAL SCLEROSIS | 55 | M | Caucasian |
| ND09956 | AMYOTROPHIC LATERAL SCLEROSIS | 63 | F | Caucasian |
| ND09995 | AMYOTROPHIC LATERAL SCLEROSIS | 34 | F | African American |
| ND10014 | AMYOTROPHIC LATERAL SCLEROSIS | 68 | M | Caucasian |
| ND10068 | AMYOTROPHIC LATERAL SCLEROSIS | 31 | M | Caucasian |
| ND10247 | AMYOTROPHIC LATERAL SCLEROSIS | 76 | M | Caucasian |
| ND10284 | AMYOTROPHIC LATERAL SCLEROSIS | 49 | M | Caucasian |
| ND10294 | AMYOTROPHIC LATERAL SCLEROSIS | 48 | M | East Indian |
| ND10547 | AMYOTROPHIC LATERAL SCLEROSIS | 62 | M | Caucasian |
| ND10588 | AMYOTROPHIC LATERAL SCLEROSIS | 38 | F | Caucasian |
| ND10734 | AMYOTROPHIC LATERAL SCLEROSIS | 42 | F | Caucasian |
| ND10735 | AMYOTROPHIC LATERAL SCLEROSIS | 51 | F | Caucasian |
| ND10741 | AMYOTROPHIC LATERAL SCLEROSIS | 34 | F | Caucasian |
| ND10905 | AMYOTROPHIC LATERAL SCLEROSIS | 58 | F | Caucasian |
| ND10912 | AMYOTROPHIC LATERAL SCLEROSIS | 31 | F | Caucasian |
| ND10966 | AMYOTROPHIC LATERAL SCLEROSIS | 64 | M | Caucasian |
| ND10985 | AMYOTROPHIC LATERAL SCLEROSIS | 45 | F | Caucasian |
| ND11252 | AMYOTROPHIC LATERAL SCLEROSIS | 47 | M | Hispanic |
| ND11411 | AMYOTROPHIC LATERAL SCLEROSIS | 53 | M | Caucasian |
| ND11506 | AMYOTROPHIC LATERAL SCLEROSIS | 49 | F | Caucasian |
| ND11524 | AMYOTROPHIC LATERAL SCLEROSIS | 59 | M | Hispanic |
| ND11548 | AMYOTROPHIC LATERAL SCLEROSIS | 67 | M | Caucasian |
| ND11554 | AMYOTROPHIC LATERAL SCLEROSIS | 57 | F | Caucasian |
