# Supplementary material for: Novel Mutations in TARDBP (TDP-43) in Patients with Familial Amyotrophic Lateral Sclerosis
Source: PLoS Genet. 2008 Sep 19;4(9):e1000193. doi: 10.1371/journal.pgen.1000193 (PMC2527686; doi:10.1371/journal.pgen.1000193)
Supplement: Table S4 — TARDBP PCR and sequencing primers. (0.03 MB DOC) [file pgen.1000193.s004.doc]

**Table S4. *TARDBP*** PCR and sequencing primers.

|  | **Primers** | **Size in bp** | **T_m_ in ^o^C** |
| --- | --- | --- | --- |
| **Exon 0 (non-coding)** | F: GAGGGAGGAGAAGACGCACTAGG  R: GCTTCGCTCCCACAAAATGG | 482 | 60 |
| **Exon 1 (non-coding)** | F: TTTCCAGGAGGCAGCCCGAGTC  R: AAGCTGGCAGCGTCCCTCGAAG | 519 | 55 |
| **Exon 2** | F: ATATACGAATCCAGACAAGC  R: TCTTCCAAACTTGTCTAACAC | 519 | 53 |
| **Exon 3** | F: AGCCTTTATTCTGTCCTCTAG  R: AGGCAGGAGGACAGTATG | 474 | 57-52 touchdown |
| **Exon 4** | F: CCACTGCATCCAGTTGAAACCAT  R: AACACACCCTGCCGCTATCTTTT | 341 | 57-52 touchdown |
| **Exon 5** | F: TCACTGCTATCCAAGGCGAATG  R: GTCTTGATCTCCTGACCTC | 386 | 58.5 |
| **Exon 6** | F: TTCCTCTGGCTTTAGATAAA  R: ACTACTGCCAAGAAACTTTATG  Seq1: AGCCACTATAAGAGTTATTTCC  Seq2: CAGGGTGGATTTGGTAATAG  Seq3: GACTTAGAATCCATGCTTGAG | 2126 | 55 |

Note. Primers for exons 0-5 were used for PCR and sequencing. Sequencing of exon 6 was performed with Primer Exon 6F and 3 additional primers listed with suffix ‘seq’.
