# Supplementary material for: Novel Mutations in TARDBP (TDP-43) in Patients with Familial Amyotrophic Lateral Sclerosis
Source: PLoS Genet. 2008 Sep 19;4(9):e1000193. doi: 10.1371/journal.pgen.1000193 (PMC2527686; doi:10.1371/journal.pgen.1000193)
Supplement: Table S5 — Primers and probes for TARDBP copy-number analyses. (0.03 MB DOC) [file pgen.1000193.s005.doc]

**Table S5. Primers and probes for *TARDBP* copy-number analyses.**

| **Exon** | **F-primer** | **R-primer** | **FAM probe** |
| --- | --- | --- | --- |
| ***TARDBP* exon 2** | CGGTTACAGCCCAGTTTCCA | CCGGACACCTCTCATACACTGA | TTCGCTACAGGAATCC |
| ***TARDBP* exon 4** | GTCACAGCGACATATGATAGATGGA | GCACAGACGCAAGTACCTTAGAATT | AAGTTTGCAGTCACACCATC |
| ***TARDBP* exon 6** | TGGTGCGTTCAGCATTAATCCA | ATGCCCATCATACCCCAACTG | CCCAGGCAGCACTACAGA |
| ***PSEN2* exon 5** | CCTTCTCCCTCAGCATCTACAC | GTGTTCAGCACGGAGTTGAG | ATTCACTGAGGACACACCC |
