# Supplementary material for: Novel Mutations in TARDBP (TDP-43) in Patients with Familial Amyotrophic Lateral Sclerosis
Source: PLoS Genet. 2008 Sep 19;4(9):e1000193. doi: 10.1371/journal.pgen.1000193 (PMC2527686; doi:10.1371/journal.pgen.1000193)
Supplement: Table S6 — Detailed Information on TARDBP Taqman genotyping assays. (0.03 MB DOC) [file pgen.1000193.s006.doc]

**Table S6. Detailed Information on *TARDBP*** Taqman genotyping assays.

|  | **Primers** | **Reporter sequences (Dye)** | **Assay** |
| --- | --- | --- | --- |
| ***TARDBP*_M337V_** | F: GCAGCACTACAGAGCAGTTG  R: ACCCGATGGGCCTGACT | CATGCCCATCATACC (VIC)  CATGCCCACCATACC (FAM) | Reverse |
| ***TARDBP*_N345K_** | F: GCAGCACTACAGAGCAGTTG  R: GCATGTTGCCTTGGTTTTGGTTATT | CCTGACTGGTTCTGC (VIC)  CCTGACTGTTTCTGC (FAM) | Reverse |
| ***TARDBP*_I383V_** | F: GCCTTCGGTTCTGGAAATAACTCTTA  R: CCCGACCCTGCATTGGAT | CCAACCAATTGCTGC (VIC) CCAACCAACTGCTGC (FAM) | Reverse |
